# COX-2 strengthens the effects of acid and bile salts on human esophageal cells and Barrett esophageal cells

**DOI:** 10.1186/s12860-022-00418-5

**Published:** 2022-04-12

**Authors:** Shen Jiangang, Kang Nayoung, Wang Hongfang, Li Junda, Chen Li, Bai Xuefeng, Li Mingsong

**Affiliations:** 1 Guangdong Provincial Key Laboratory of Gastroenterology, Institute of Gastroenterology of Guangdong Province, Department of Gastroenterology, Nanfang Hospital, Southern Medical University, Guangzhou, 510515 China; 2 Department of Gastroenterology, Shenzhen Longhua District People’ Hospital, Shenzhen, 518109 China; 3 Department of Gastroenterology, Shenzhen Luohu People’ Hospital, Shenzhen, 518003 China; 4 Department of Pathology and Comprehensive Cancer Center, Ohio State University Medical Centre, 129 Hamilton Hall, 1645 Neil Avenue, Columbus, OH 43210 USA

**Keywords:** Barrett’s esophagus, Esophageal adenocarcinoma, Cyclooxygenase-2, Nuclear factor kappa B, Caudal-related homeobox transcription factor-2, Bone morphogenic protein-4

## Abstract

**Aims:**

Investigate the effect and mechanism of COX-2 on viability, intestinal metaplasia, and atypia in human esophageal squamous and Barrett esophageal cell lines.

**Methods:**

Human esophageal squamous and Barrett esophageal cell lines were transfected with a COX-2 expression vector and a COX-2 siRNA, and then were treated with acid, bile salts, and a mixture of both. Cell viability, the expression of COX-2, NF-κB(p65), CDX-2, MUC2, c-myb, and BMP-4, and the morphology and microstructure of cells were then observed.

**Results:**

The viability of COX-2 overexpressed cells was significantly higher than that of control cells, while the viability of COX-2 siRNA-treated cells was significantly lower than that of control cells. Intestinal metaplasia and atypia were observed in cells overexpressing COX-2. Acid, bile salts, and their mixture inhibited the viability of these two cell lines, but the inhibitory effect of the mixture was stronger than a single treatment in either. SiRNA mediated knockdown of COX-2 strengthened the antiproliferative effects of the mixture on HET-1A and BAR-T cells. The expression of p-p65, CDX-2, and BMP-4 was positively correlated with COX-2 expression, while the expression levels of p65, MUC2, and c-myb remained unchanged.

**Conclusion:**

COX-2 may influence the viability, atypia, and intestinal metaplasia of human esophageal cells and Barrett esophageal cells. Activation of the p-p65, CDX-2, and BMP-4 signaling pathways by COX-2 may be part of this mechanism.

**Supplementary Information:**

The online version contains supplementary material available at 10.1186/s12860-022-00418-5.

## Introduction

Barrett’s esophagus (BE) is characterized by the replacement of normal squamous epithelium (SQ) by intestinal-type columnar epithelium in the distal esophagus. BE is predominantly relevant to gastroesophageal reflux disease (GERD), genetics, obesity, lifestyle, gender, and race, among which GERD is the most important [1]. Gastroesophageal reflux, mainly acid and bile, plays an important role in the occurrence of BE [2, 3]. BE is a precancerous lesion of esophageal adenocarcinoma (EAC) [4]. EAC has a poor prognosis, with a 5-year survival rate of 83% to 90% after early diagnosis, compared with 10% to 15% at later stage [5]. Early EAC is usually asymptomatic and is not noticed until a local invasion occurs. Despite recent improvements and improvements in surveillance and treatment, the 5-year overall survival rate of EAC remains the lowest among all cancers [6]. Early diagnosis and active treatment of BE and EAC will help to reduce the morbidity and mortality of EAC. Whether BE is derived from esophageal squamous cells or stem cells remains controversial, but most studies have suggested that BE may be derived from esophageal squamous cells and EAC is derived from BE. Currently, the mechanism by which BE occurs and develops into EAC is wholly unknown [7–9].

Cyclooxygenase-2 (COX-2) is a key enzyme for the initiation of inflammatory responses [10, 11], and it also participates in the initiation and development of a variety of inflammatory conditions by promoting cell proliferation, inhibiting apoptosis, promoting angiogenesis, increasing the invasion ability of tumor cells, and inhibiting the immune function of the body [12–15]. It was found that COX-2 expression in human BE tissues was significantly higher than that in surrounding squamous cells and control tissues [16–18] and was significantly higher in EAC tissues [19], suggesting that COX-2 may be involved in the occurrence and development of BE. Previous studies have also shown that the high expression of toll-like receptor 4 (TLR-4) in BE can promote the strong expression of COX-2 and lead to the transformation of BE cells into atypia [20]. COX-inhibitors such as indomethacin can inhibit the growth of esophageal adenocarcinoma in nude mice and induce its regression [21]. These studies suggest that COX-2 may also play an important role in the occurrence of BE and EAC, but the effect of COX-2 regulation on biological behavior of esophageal squamous cell and BE cells in human has not been reported. Some studies have used celecoxib, a specific COX-2 inhibitor, to interfere with human BE and EAC, but the response is not satisfactory. The reason for the poor efficacy of COX-2 inhibitors in the treatment of BE and EAC may be acid and / or bile reflux, which is not effectively cleared during treatment [22].

In this study, single cell lines were used to investigate the role of COX-2 in BE genesis and atypia, avoiding many interference factors. Gene transfection is an efficient method to regulate the expression of target genes with high efficiency and little interference. We aimed to determine the effect of COX-2 on viability, intestinal metaplasia, and atypia in a normal esophageal squamous cell line (HET-1A) and a Barrett esophageal cell line (BAR-T). To determine the mechanisms driving these, we also tested the expression of cytokines associated with BE and EAC, such as nuclear factor kappa B (NF-κB), bone morphogenetic protein-4 (BMP-4), caudal-related homeobox transcription factor-2 (CDX-2), muc-2, and c-myb. To further verify our hypothesis, we simulated the clinical tissue microenvironment and investigated whether COX-2 could reverse the behavioral effects of acid, bile salts and their mixtures on HET-1A and BAR-T cells by inhibiting COX-2 expression.

## Materials and methods

### Reagents

Mouse anti-human monoclonal antibodies and fluorescently labeled sheep anti-mouse secondary antibody against COX-2, BMP-4, NF-κB (p65), p-p65, CDX-2, muc-2, c-myb, and GAPDH were all purchased from Epitomics,USA. MTT and DMSO were purchased from Sigma. RNA extraction TRIzol kit, Lipofectamine 2000, RPMI1640, and fetal bovine serum were purchased from Invitrogen, USA. The bile salts media contained a mixture of conjugated bile salts, including glycocholic acid, taurocholic acid, glycochenodeoxycholic acid, taurochenodeoxycholic acid, glycodeoxycholic acid, and taurodeoxycholic acid (Sigma) in a 20:3:15:3:6:1 molar concentration as previously described [23]. The acidified medium was titrated using HCl (Sigma). When used, a specific volume of medium was adjusted to the required concentration.

### Cell culture

The human esophageal squamous cell line HET-1A was obtained from American Type Culture Collection (ATCC, Manassas, VA, USA). HET-1A is a normal human esophageal epithelial cell line immortalized by transfection of the SV40 T antigen early region gene [24]. BAR-T is a human Barrett’s esophagus cell line. HET-1A and BAR-T cells were cultured as previously described [25].

### Cell proliferation assays

Cell proliferation was analyzed using the 3-(4,5-dimethylthiazol-2-yl)-2,5 -diphenyltetrazolium bromide assay, according to the manufacturer’s instructions as described previously [26].

### COX-2 siRNA and COX-2 expression plasmid transfection of HET-1A and BAR-T cells

COX 2 siRNAs and COX-2 expression plasmid were acquired from Santa Cruz Biotechnology. Each siRNA was transfected into cells using Lipofectamine reagent according to the manufacturer's protocol. When HET-1A cells grew to 90% confluence, they were digested, centrifuged, re-suspended, and transferred to a 24-well plate. Transfection was performed when the cells grew to about 60%–70% confluence. The experiment was divided into four groups: a blank control group (HET-1A/BAR-T cells without any treatment), a negative control group (HET-1A/BAR-T cells transfected with negative siRNA), a COX-2 group (HET-1A/BAR-T cells transfected with COX-2 overexpression plasmid), and a COX-2 siRNA group (HET-1A/BAR-T cells transfected with COX-2 siRNA). Western blot was used to detect COX-2 expression to determine transfection efficiency.

### Western blot analysis

Cells were inoculated in 6-well plates, cultured for 48 h, collected, and washed on ice with PBS three times. Cells were then lysed using 1% Triton X-100. Total protein was collected and SDS-PAGE gel electrophoresis and membrane transfer were performed. The primary antibodies used were mouse antihuman COX-2, CDX-2, BMP-4, p-p65, muc-2, c-myb, and GAPDH, and the secondary antibody was a sheep anti-mouse antibody labeled with a fluorophore. The fluorophore signals were visualized using ECL reagent (Tanon, China). Fluorescence was measured and the gray value of the reaction band was determined by ImageJ software. GAPDH served as an internal reference, and the relative value was computed. These experiments were repeated three times.

### Acid and bile salts exposure of HET-1A and BAR-T cells

For individual experiments, cells were cultured in one of four different experimental media: 1) control medium that consisted of neutral full growth medium (pH 7.0); 2) neutral bile salts medium (containing conjugated bile acid at a total concentration of 500 μM at pH 7.0); 3) acidic rich growth medium (brought to a pH of 6.0 with HCl); and 4) acidic bile salts medium (the same bile acid solution at pH 6.0). The medias were added for 10 min to equally seeded wells of cells, then removed and replaced with a neutral pH medium until the next treatment. HET-1A and BAR-T cells were treated with either experimental or control medium 3 times per day for 7 days (unless otherwise stated).

### Electron microscopy

Transmission electron microscopy (TEM) was used to detect ultrastructural changes in HET-1A and BAR-T cells. Cells were attached with 3% glutaraldehyde in 0.1 mM cacodylate buffer. Samples were then fixed using 1% osmium tetroxide, dehydrated in a graded series of ethanols, and integrated into epoxy resin. Ultrathin sections were measured for morphological changes using a Japan Electron Optics Laboratory JEM-2010 transmission electron microscope.

### Statistical analysis

SPSS 19.0 statistical software was used for statistical analysis, and all measurement data are presented as the mean ± standard deviation (SD). The methods used were factorial analysis of variance, repeated measurement analysis of variance, and one-way analysis of variance (ANOVA). The least significant difference (LSD) method was used for multiple comparisons between groups. Results were statistically significant at *P* < 0.05.

## Results

### Effects of overexpression or gene silencing of COX-2 on the proliferation and morphology of HET-1A and BAR-T cells

**Fig. 1:**
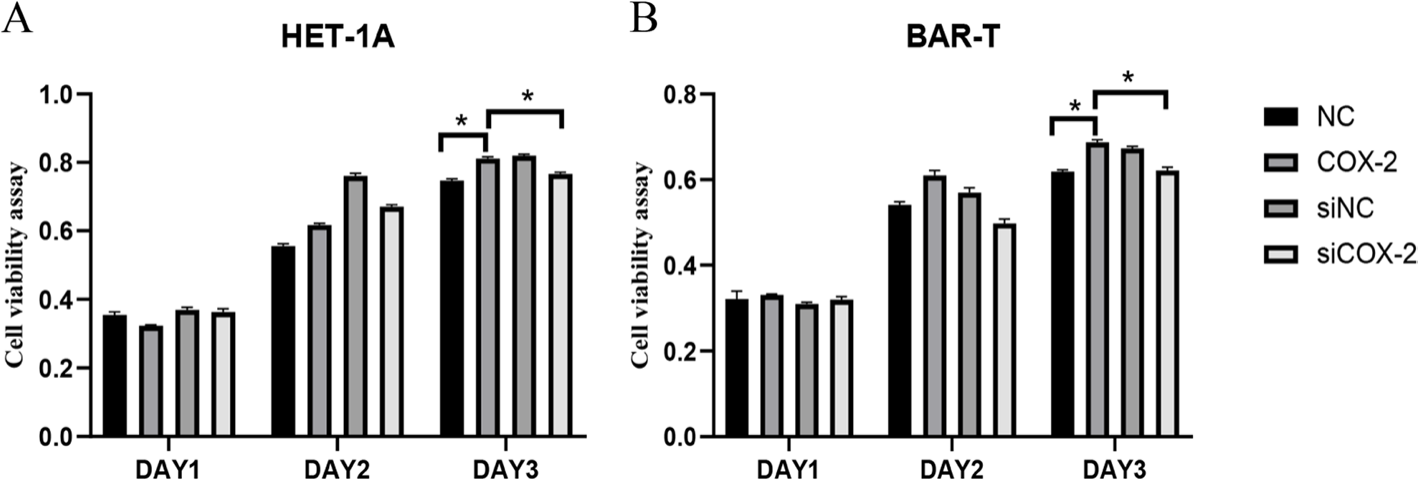
The cell viability was detected by MTS. **A** The overexpression and gene silencing of COX-2 in HET-1A cells. **B** The overexpression and gene silencing of COX-2 in BAR-T cells. The data are shown as the mean ± SD. *p < 0.05

**Fig. 2:**
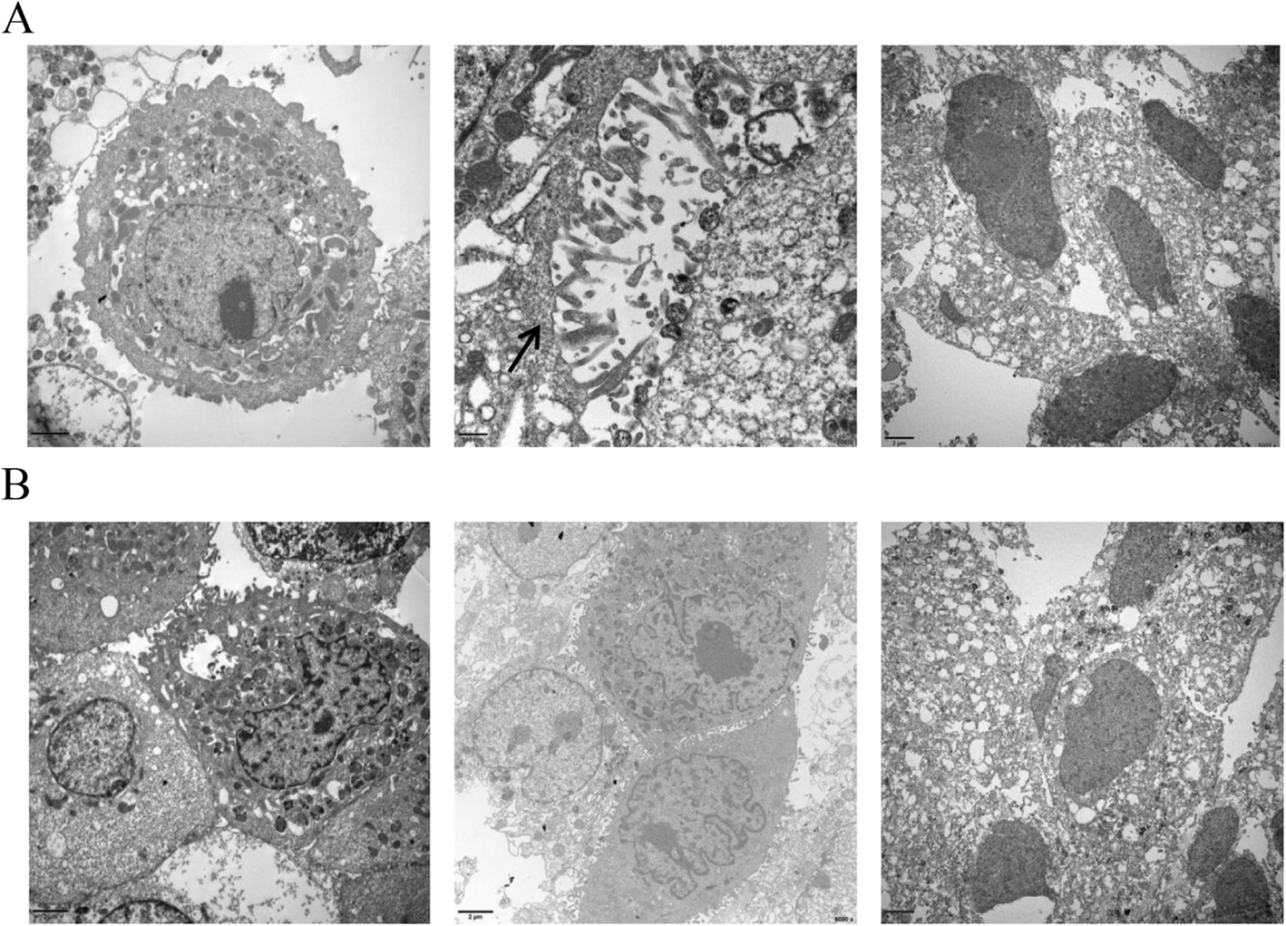
The morphology of cells were observed by electron microscopy. **A** The three images are the negative control group, the COX-2 overexpression group, and the COX-2 siRNA group in HET-1A cells for 3 days. The arrow is adenoid cavity structures. **B** The three images are the negative control group, the COX-2 group, and the COX-2 siRNA group in BAR-T cells for 3 days. The arrow is autophagosomes

Cell proliferation was assessed by MTS, as shown in Fig. 1. On the third day after overexpression or silencing COX-2, the proliferation rate of the COX-2 group was significantly higher than that of the control group (*P* < 0.05) in HET-1A and BAR-T cells, while the cellular proliferation of the siCOX-2 group was significantly lower than that of the COX-2 group (*P* < 0.05). Meanwhile, when COX-2 was overexpressed in HET-1A cells for 3 days, an increase in the number of microvilli on the cell surface was observed by electron microscopy, and adenoid cavity structures were observed, suggesting intestinal metaplasia of the cells, while siRNA of COX-2 showed no such intestinal metaplasia. Nuclear abnormalities and autophagosomes were observed after COX-2 overexpression in BAR-T cells for 3 days, suggesting atypia of these cells, while siRNA of COX-2 induced no such changes (Fig. 2A and B).

### Effects of COX-2 overexpression and gene silencing on COX-2, CDX-2, BMP-4, p-p65, p65, muc-2, and c-myb in HET-1A and BAR-T cells

**Fig. 3:**
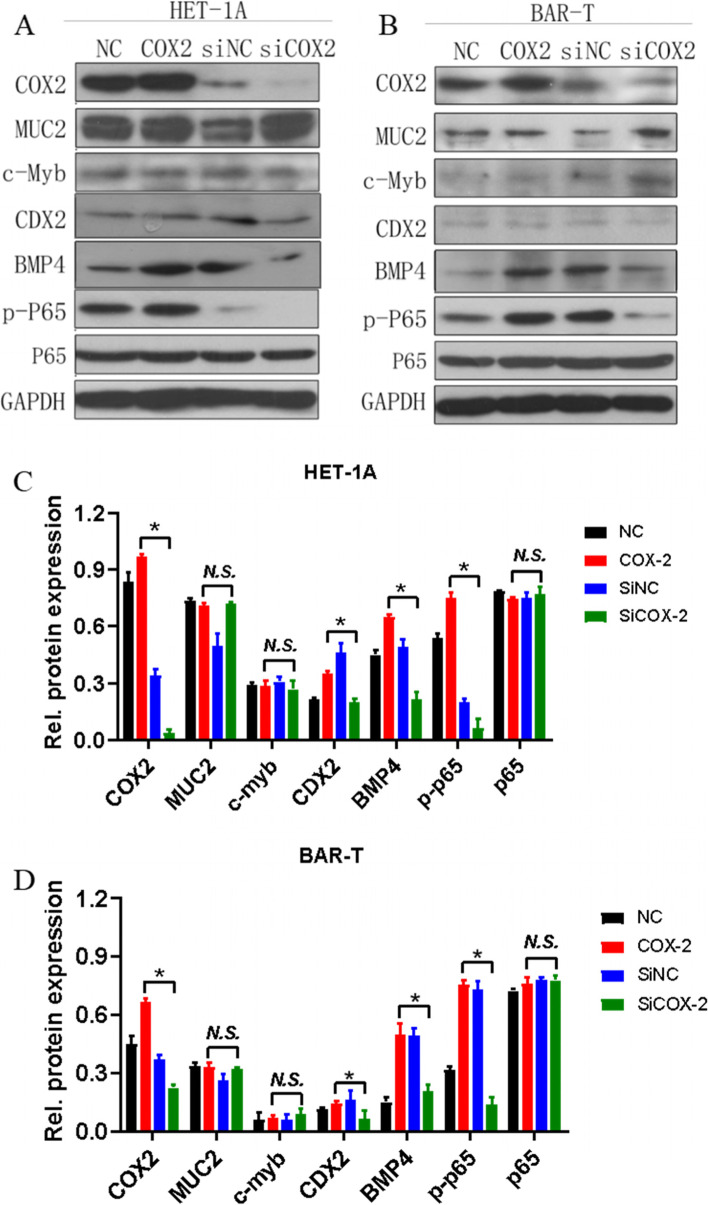
The expression levels of COX-2, CDX-2, BMP-4, p-p65, p65, muc-2, and c-myb were determined by western blot. **A, C** The expression levels of related proteins in HET-1A cells and quantitative analysis. **B, D** The expression levels of related proteins in BAR-T cells and quantitative analysis. The data are shown as the mean ± SD (*n* = 3). *p < 0.05, N.S, no significant effect

Protein expression levels of COX-2, p65, p-p65, CDX-2, and BMP-4 were next assessed by western blot on the second day after COX-2 overexpression or gene silencing in these two cell lines. As shown in Fig. 3, COX-2 overexpression or knockdown effects were significant in both cell lines. The expression levels of BMP-4, p-p65, and CDX-2 were all positively correlated with COX-2 expression changes, while the expression levels of p65, MUC2, and c-myb remained unchanged.

### Effects of acid, bile salts, and their mixture on the proliferation of HET-1A and BAR-T cells

**Fig. 4:**
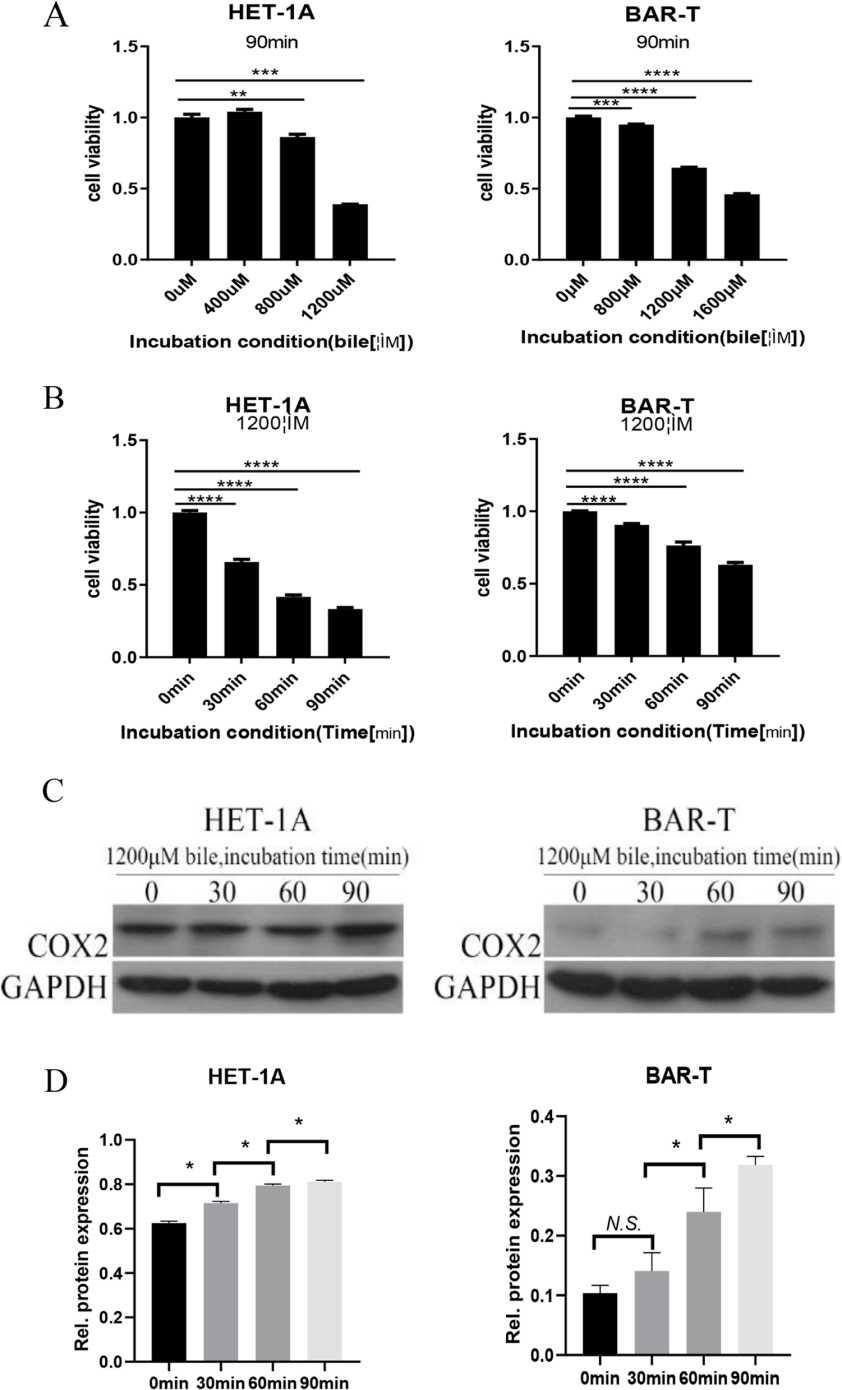
Effects of bile salts on the proliferation of HET-1A and BAR-T cells. **A** After 90 min, cell proliferation was assessed by MTS in different concentrations of bile salts in HET-1A and BAR-T cells. **B** At a concentration of 1200 μmol/L of bile salts, cell proliferation was assessed by MTS at different times in HET-1A and BAR-T cells. **C, D** At a concentration of 1200 μmol/L of bile salts, the expression levels of COX-2 were determined by western blot at different times in HET-1A and BAR-T cells. The data are shown as the mean ± SD. *p < 0.05, **p < 0.01, ***p < 0.001, N.S, no significant effect

Different concentrations of bile salts were tested on both cell lines (HET-1A: 0 μmol/L, 400 μmol/L, 800 μmol/L, and 1200 μmol/L; and BAR-T: 0 μmol/L, 800 μmol/L, 1200 μmol/L, and 1600 μmol/L). MTS test results are shown in Fig. 4A. In this experiment, bile salts concentrations of 1200 μmol/L were selected for both cells, and the treatment time was set at 0, 30, 60, or 90 min for the experiment presented in Fig. 4B. A concentration of 1200 μmol/L of bile salts was selected, and the treatment time was set as 30 min, 60 min, or 90 min to determine the COX-2 protein expression levels. The detection results are presented in Fig. 4C and D. When HET-1A cells were treated with bile salts for 90 min and BAR-T cells were treated with bile salts for 60 min and 90 min, the COX-2 expression was substantially upregulated.

**Fig. 5:**
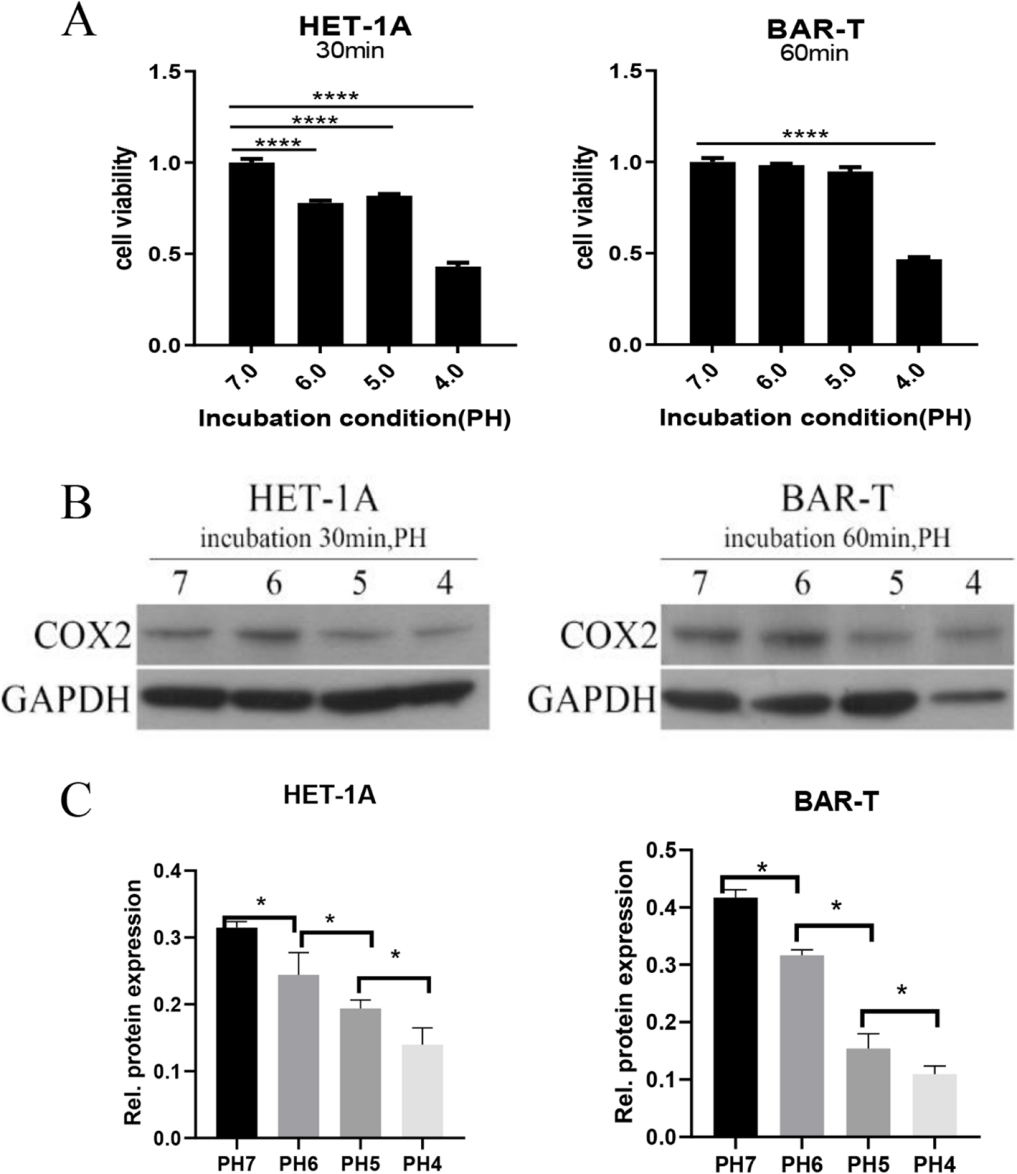
Effects of acid on the proliferation of HET-1A and BAR-T cells. **A** After 30-min treatment of HET-1A cells and 60-min treatment of BAR-T cells, cell proliferation was assessed by MTS in different concentrations of hydrochloric acids. **B, C** After 30-min treatment of HET-1A cells and 60-min treatment of BAR-T cells, the expression levels of COX-2 were determined by western blot in different concentrations of hydrochloric acids. The data are shown as the mean ± SD. *p < 0.05, **p < 0.01, ***p < 0.001, N.S, no significant effect

Next, the pH value of the medium was adjusted with hydrochloric acid, and the cells were cultured in medium with pH values of 4.0, 5.0, or 6.0. In the blank control group, the medium was not treated with hydrochloric acid. Cell viability was then measured by MTS, as shown in Fig. 5A, and the COX-2 protein expression level was measured by western blot, as shown in Fig. 5B and C. We found that after incubation with hydrochloric acid at pH 6.0, 5.0, or 4.0 for specific length time, the cell activity and COX-2 expression were both upregulated. Based on the above experimental results, a pH of 6.0 was selected for the treatment of both cell lines, with HET-1A cell being treated for 30 min and BAR-T cell being treated for 60 min.

**Fig. 6:**
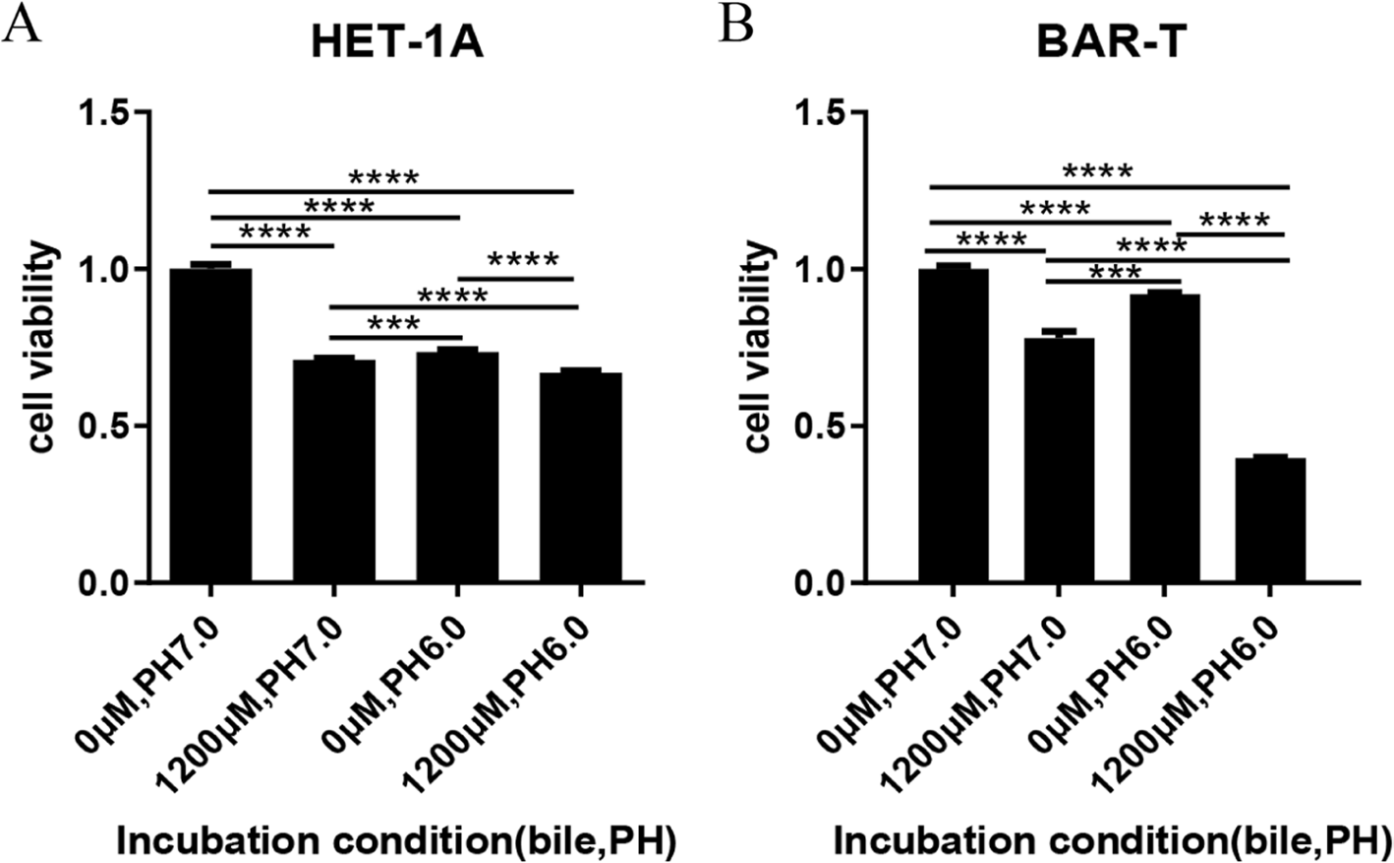
Effects of acid, bile salts, and their mixture on the proliferation of HET-1A and BAR-T cells. **A** After 30-min treatment of HET-1A cells, cell proliferation was detected by MTS in the four groups. **B** After 60-min treatment of BAR-T cells, cell proliferation was detected by MTS in the four groups. The data are shown as the mean ± SD. ***p < 0.001

Based on the above experimental results, four groups (bile salts and hydrochloric acid) were made, namely, a control group (0 μmol/L, pH 7), a bile salts group (1200 μmol/L, pH 7), a hydrochloric acid group (0 μmol/L, pH 6), and a hydrochloric acid and bile salts mixed group (1200 μmol/L, pH 6) to determine if the effect on COX-2 was due to the presence of bile salts or the change in pH. Cell proliferation was measured by MTS after a 30 min treatment in HET-1A cells and a 60 min treatment in BAR-T cells. As shown in Fig. 6, acid, bile salts and the mixture of both inhibited the proliferation of these two cell lines, but the inhibitory effect of bile salts + hydrochloric acid was stronger than bile salts or hydrochloric acid treatments alone.

### Effects of acid, bile salts, and their mixture on COX-2, CDX-2, BMP-4, and p-p65 expression in HET-1A and BAR-T cells

**Fig. 7:**
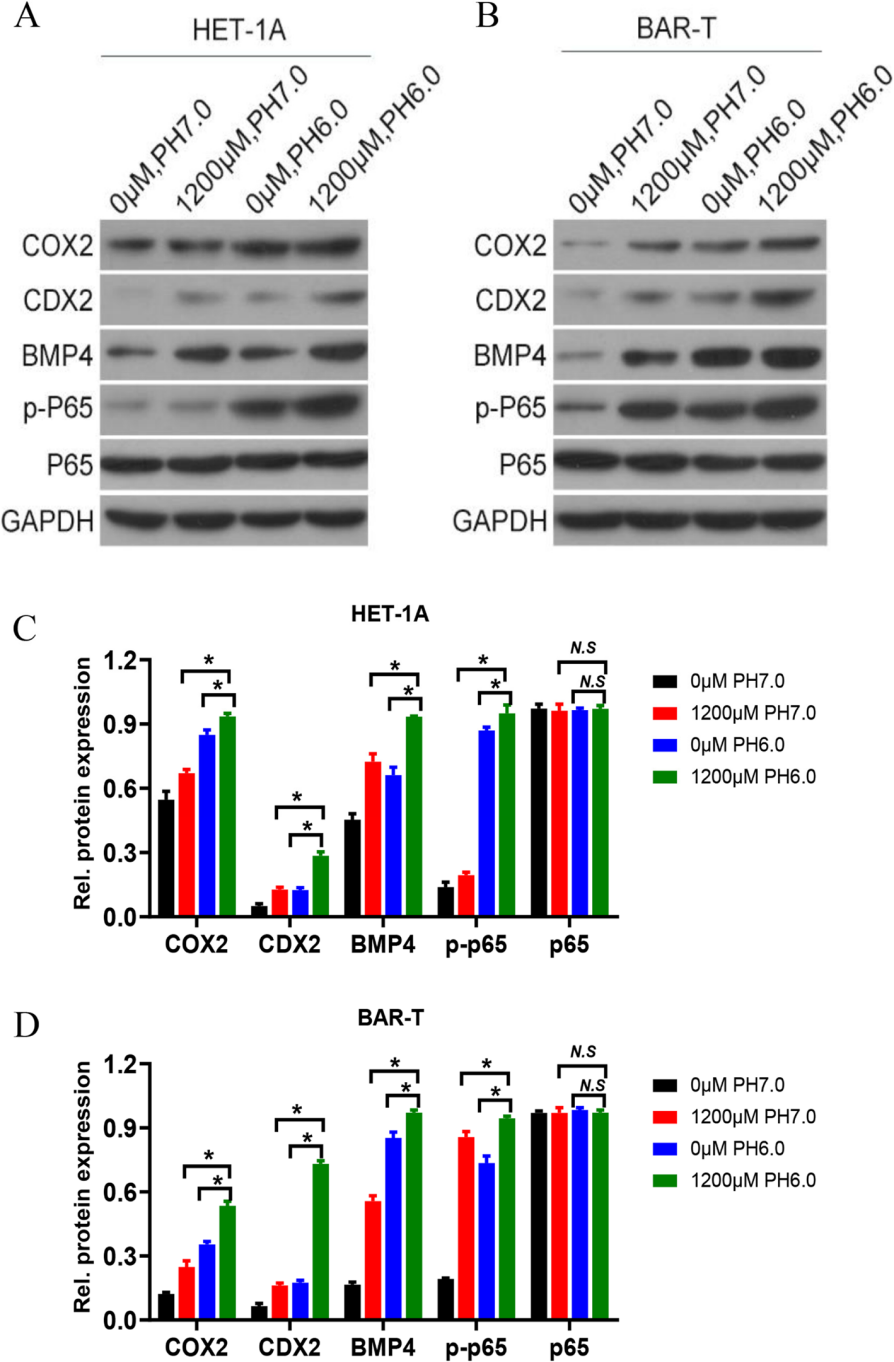
Effects of acid, bile salts, and their mixture on COX-2, CDX-2, BMP-4, and p-p65 expression in HET-1A and BAR-T cells. **A, C** The expression levels of related proteins in HET-1A cells and quantitative analysis. **B, D** The expression levels of related proteins in BAR-T cells and quantitative analysis. The data are shown as the mean ± SD (*n* = 3). *p < 0.05, N.S, no significant effect

To further explore the findings in the above groups, the protein expression levels of COX-2, p65, p-p65, CDX-2, and BMP-4 were assessed after 30 min treatment of HET-1A cells and 60 min treatment of BAR-T cells. As shown in Fig. 7, the protein expressions of COX-2, CDX-2, BMP-4, and p-p65 in each group were increased compared with the normal group, and the expression of these proteins in the bile salts and hydrochloric mixed group was the strongest. However, the expression of p65 was unchanged in all groups.

### Effects of COX-2 gene silencing on the proliferation of HET-1A and BAR-T cells after acid and bile salts treatment

**Fig. 8:**
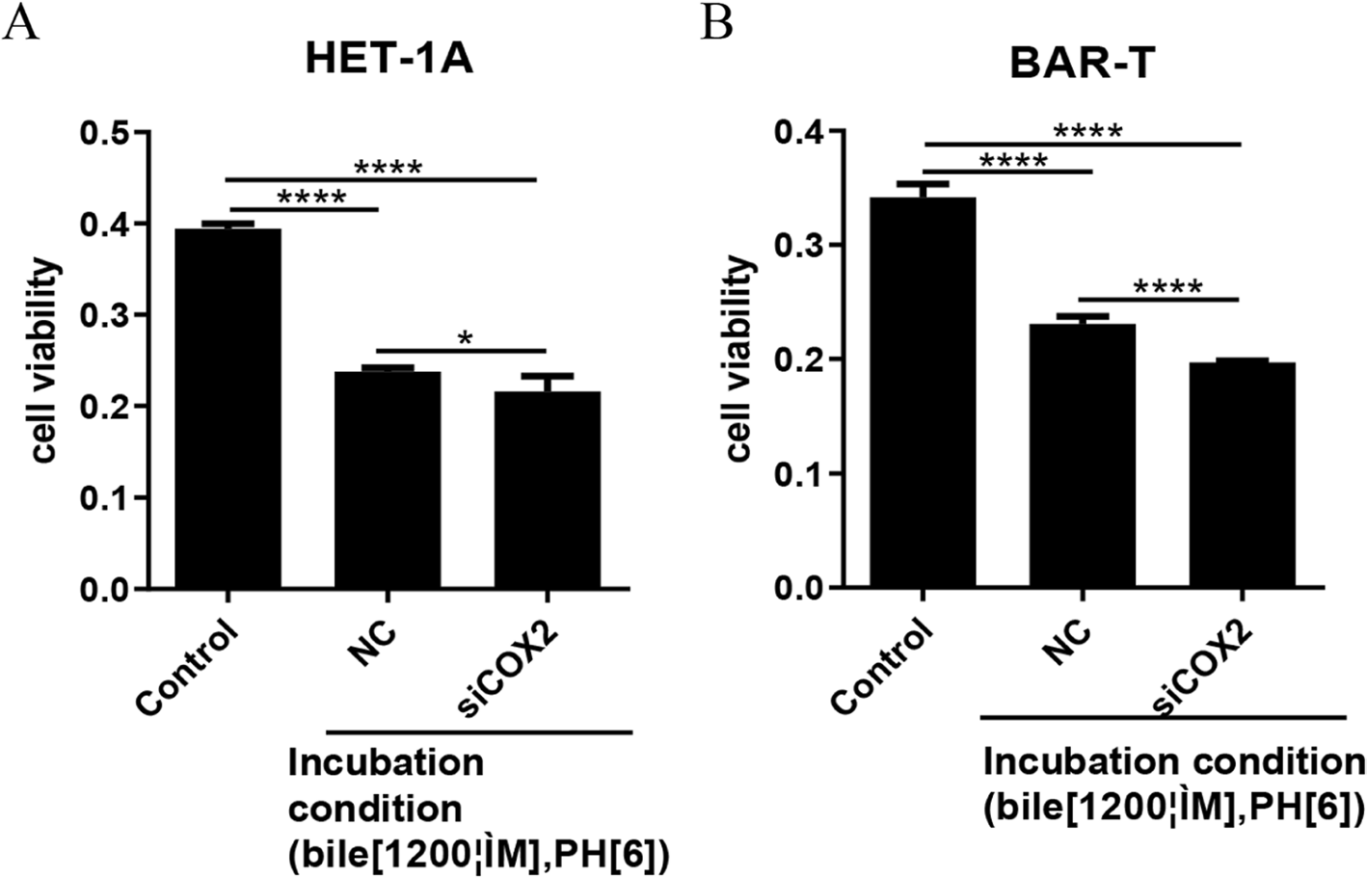
Effects of COX-2 gene silencing on the proliferation of HET-1A and BAR-T cells after acid and bile salts treatment. **A** After acid and bile salts treatment and COX-2 gene silencing, cell proliferation was detected by MTS in HET-1A cells. **B** After acid and bile salts treatment and COX-2 gene silencing, cell proliferation was detected by MTS in BAR-T cells. The data are shown as the mean ± SD. *p < 0.05, ****p < 0.001

Next, these cells were transfected with COX-2 siRNA, and treated with hydrochloric acid pH 6.0 and bile salts at 1200 μmol/L for 48 h before sample collection. HET-1A cells were treated for 30 min and BAR-T were treated for 60 min. Cell proliferation was detected by MTS, and the results are presented in Fig. 8A and B. In response to acid and bile salts, multiple inflammatory factors are activated and inhibit cell proliferation, which exceeds the effect of COX-2 on cell proliferation. After silencing COX-2 expression, the promoting effect of COX2 on cell proliferation was inhibited, and cell proliferation was further inhibited. So COX-2 siRNA silencing further enhanced the inhibitory effect of the acid and bile salts mixture on the proliferation of HET-1A and BAR-T cells.

### Effects of COX-2 gene silencing on expression of COX-2, CDX-2, BMP-4, and p-p65 in HET-1A and BAR-T cells after acid and bile salts treatment

**Fig. 9:**
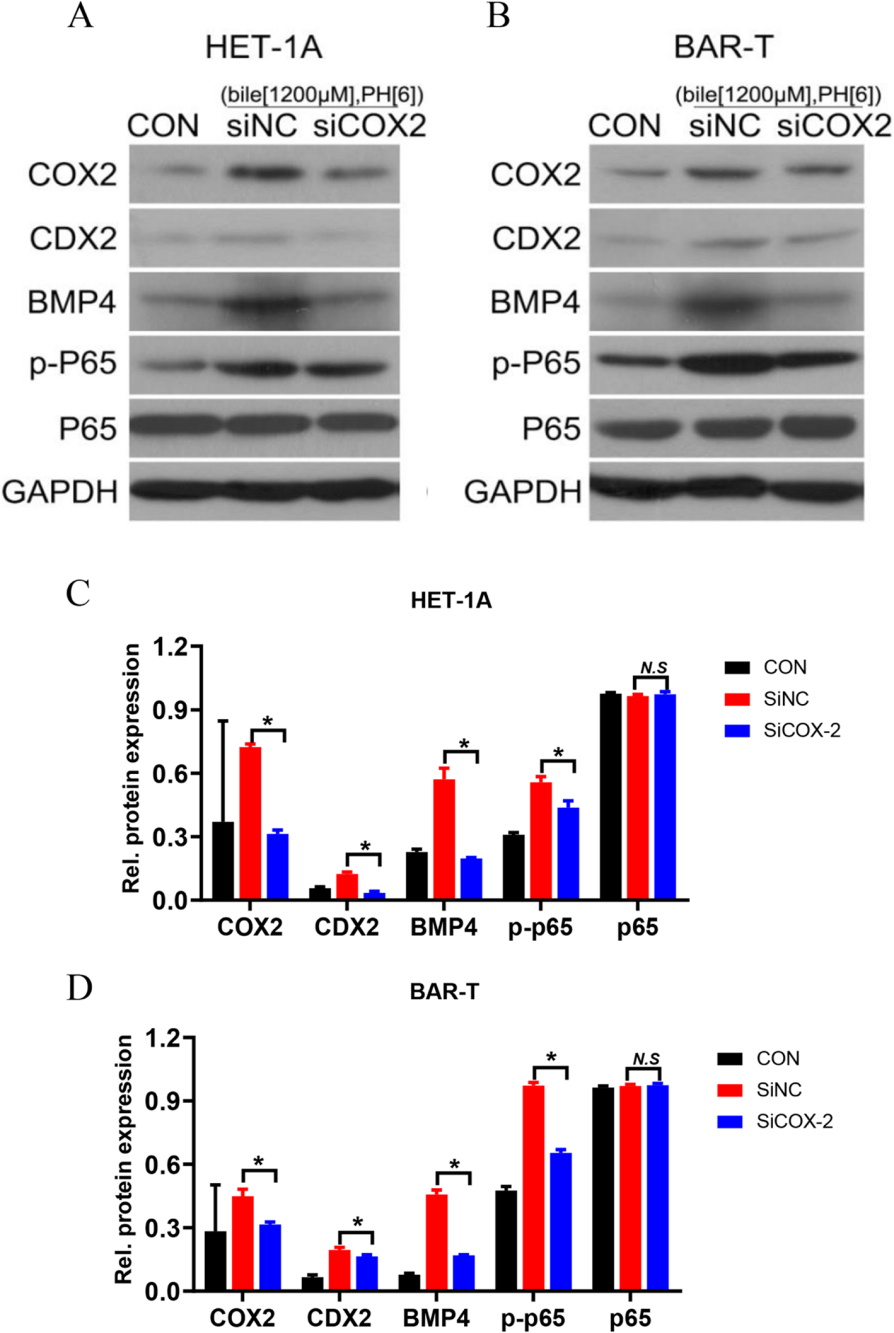
Effects of COX-2 gene silencing on expression of COX-2, CDX-2, BMP-4, and p-p65 in HET-1A and BAR-T cells after acid and bile salts treatment. **A, C** The expression levels of related proteins in HET-1A cells and quantitative analysis. **B, D** The expression levels of related proteins in BAR-T cells and quantitative analysis. The data are shown as the mean ± SD (*n* = 3). *p < 0.05, N.S, no significant effect

Cells were transfected with COX-2 siRNA, and treated with hydrochloric acid (pH 6.0) and bile salts (1200 μmol/L) for 48 h before sample collection. HET-1A cells were treated for 30 min and BAR-T cells were treated for 60 min. Next, the protein expression levels of COX-2, p65, p-p65, CDX-2, and BMP-4 were determined using western blot. As shown in Fig. 9, the expressions of COX-2, CDX-2, BMP-4, and p-p65 proteins were upregulated after treatment with acid and bile salts mixture, while they were downregulated after COX-2 siRNA was transfected. The expression of p65 was not changed.

### Effects of acid and bile salts mixture on the morphology of HET-1A and BAR-T cells before and after COX-2 gene silencing

**Fig. 10:**
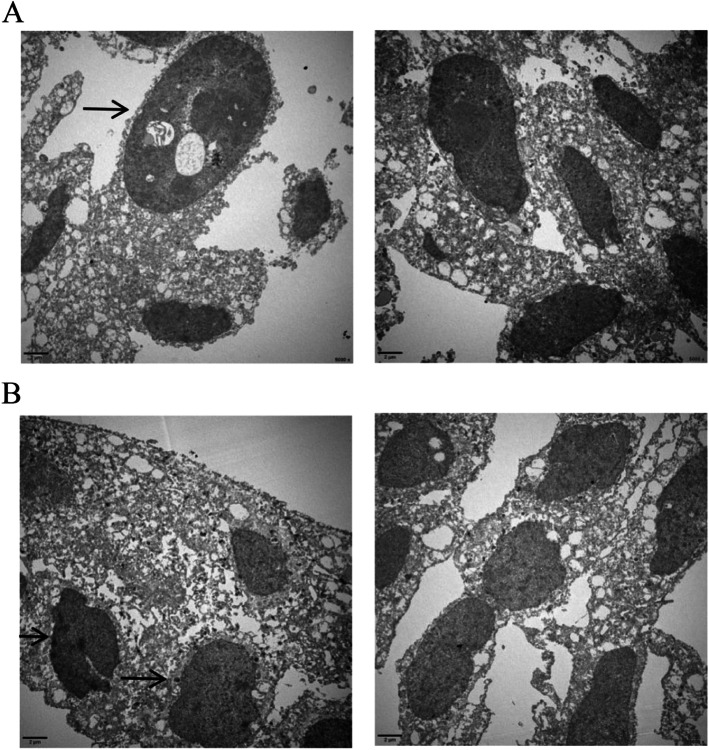
Effects of acid and bile salts mixture on the morphology of HET-1A and BAR-T cells before and after COX-2 gene silencing. **A** The images are from the mixture of acid and bile salts groups before and after COX-2 gene silencing in HET-1A cells. **B** The images are from the mixture of acid and bile salts groups before and after COX-2 gene silencing in BAR-T cells

After the mixture of acid and bile salts acted on the cells, nuclear inclusion bodies, autophagosome-like structures, and other cellular morphological manifestations were observed in HET-1A cells. Owing to the damage induced by acid and bile salts, changes such as incomplete capsule, formation of vacuolar structures in the cytoplasm, mitochondrial swelling, cavitation, and disappearance of the chute, and the intestinal metaplasia of the cells were not obvious. Heteromorphic changes such as nuclear heteromorphism was found in BAR-T cells. After gene silencing of COX-2 followed by treatment with a mixture of acid and bile salts, no such changes were seen in these two cell lines (Fig. 10A and B).

## Discussion

In this study, we showed that overexpression of COX-2 in HET-1A cells could promote cell proliferation, accompanied by intestinal metaplasia, while COX-2 siRNA could inhibit cell proliferation and prevent the emergence of intestinal metaplasia. In BAR-T cells, overexpression of COX-2 could promote cell proliferation, accompanied by cellular heteromorphism, while COX-2 siRNA could inhibit cell proliferation and the development of heteromorphism. These results suggest that COX-2 may play a major role in the occurrence and development of BE, which was consistent with our hypothesis.

As an essential transcription factor in inflammatory response, NF-κB is believed to play an important role in the development of cancer and participate in the apoptosis of various cells and tissues [27, 28]. Studies have found that NF-κB is increased in BE and esophageal adenocarcinoma tissues, which may play a role by activating surviving, an antiapoptotic factor [29]. Inhibition of NF-κB in esophageal squamous cells inhibits cell proliferation, accompanied by decreased COX-2 expression [30]. Inhibition of NF-κB expression in EAC cells reduces the expression of COX-2 and CDX-2, and enhances apoptosis of EAC cells [31]. The above studies suggest that NF-κB plays a major role in the occurrence and development of BE, and NF-κB acts as the upstream molecule to regulate the expression of COX-2. Park et al. found that celecoxib, a COX-2 inhibitor in leiomyoma cells, could inhibit cell proliferation through the NF-κB pathway, suggesting that COX-2 could regulate NF-κB in leiomyoma cells [32]. In this study, we found that COX-2 could regulate the expression of NF-κB in HET-1A and BAR-T cells, and phosphorylated NF-κB(p-p65) may play a significant role in the effect of COX-2 on HET-1A and BAR-T cell proliferation and cell morphology changes. Storz L et al. have demonstrated that acid can activate the phosphorylation expression of NF-κB in BE and EAC cells and reduce the in vitro chemotherapy effect of 5-FU, while PPIs can indirectly help EAC patients overcome chemotherapy resistance by restoring esophageal pH value. According to the results of this study, inhibition of COX-2 and reduction of NF-κB phosphorylation expression may help EAC patients overcome 5-FU chemotherapy resistance [33].

Bone morphogenetic proteins (BMPs) are mainly expressed in embryonic development or disease states such as cancer tissue, and its family members can participate in cell proliferation, migration, apoptosis, and differentiation [34]. Studies have shown that BMP-4 was highly expressed in BE and EAC tissues, and its downstream signaling molecule ID2 was also highly expressed, suggesting that the BMP-4 signaling pathway was activated in BE and EAC [35]. After recombinant BMP-4 treatment in vitro, normal squamous epithelial cells were shown to be transformed into columnar epithelial cells, and the intestinal epithelial markers Villin and CDX-2 were detected [36]. The BMP signaling pathway can activate SOX9 and plays an important role in the occurrence and development of BE[37]. In this study, it was shown that COX-2 could regulate the expression of BMP-4 in HET-1A and BAR-T cells, and BMP-4 may also play an important role in the effect of COX-2 on HET-1A and BAR-T cell proliferation and cell morphology.

As a member of the caudal homologous nuclear transcription factor family, CDX is a nuclear transcription factor specifically expressed during intestinal development and regulates the proliferation and differentiation of intestinal epithelial cells [38, 39]. CDX-2 plays a key role in intestinal metaplasia in BE, and its expression in the esophagus is an early explicit marker of intestinal metaplasia [40–42]. In the environment of acid or bile acid, demethylation of the promoter of CDX-2 could promote the expression of CDX-2 in esophageal epithelium and promote intestinal metaplasia [43]. CDX-2 could be involved in the carcinogenic mechanism of EAC by inhibiting the expression of DNA repair enzymes and promoting the expression of CDX-2 [44]. In this study, we found that COX-2 could regulate CDX-2 expression in HET-1A cells, suggesting that COX-2 plays a more prominent role in intestinal metaplasia in esophageal squamous cells. CDX-2 was also found to be highly expressed in digestive tract tumors and is involved in the occurrence of EAC [45, 46]. In this study, we found that COX-2 could regulate the expression of CDX-2 in BAR-T cells, suggesting a role in the occurrence and heteromorphism of BE. The study found that CDX-2 was almost completely undetectable in esophageal squamous cells [47], while in our experiments, CDX-2 was expressed at low levels in HET-1A cells, which may a result of the cell line used in our studies.

MUC2 is mainly expressed in intestinal metaplasia and malignant lesions of BE [40, 48, 49]. C-myb is an intranuclear oncogene, which is involved in cell proliferation and plays an important role in the proliferation regulation of numerous malignant tumor cells. Studies have shown that upregulation of mRNA expression of c-myb is an early event in the process of BE transition to esophageal cancer [50, 51]. However, changes in COX-2 expression in HET-1A and BAR-T cells did not cause changes in the expressions of MUC2 or c-myb, possibly because the regulation of these two proteins was not dependent on COX-2.

The above studies indicate that COX-2 plays an important role in the process of intestinal metaplasia of esophageal squamous epithelial cells and atypia in BE cells, and its mechanism appears to function through the regulation of the expression of p-p65, BMP-4, CDX-2, and other cytokines. To verify these conclusions, we used different concentrations of acid, bile salts and a mixture of the two to treat HET-1A and BAR-T cells to simulate the human microenvironment in GERD, and we assessed the role and possible mechanism of COX-2 in this process. It should be noted that the simulated conditions were lower than the pH of the physiological environment, and only one cell line was studied under each condition.

Our study showed that the proliferation of HET-1A or BAR-T cells was inhibited under the action of acid, bile salts, and the mixture of the two, and the mixture of acid and bile salts had the strongest effect. These treatments resulted in increased expressions of COX-2, BMP-4, p-p65, and CDX-2. SiRNA mediated depletion of COX-2 enhanced the inhibitory effect of acid, bile salts, and the mixture of the two on the proliferation of HET-1A and BAR-T cells, accompanied by the reduced expressions of COX-2, BMP-4, p-p65, and CDX-2. These observations suggest that in the environment of acid, bile salts, and the mixture of the two, the proliferation of HET-1A and BAR-T cells was closely related to the expression of COX-2, which further regulates cytokines such as p-p65, BMP-4, and CDX-2.

## Conclusions

COX-2 plays an important role in the occurrence and development of BE, which can be used as a target for the diagnosis and treatment of BE and EAC. Activation of the NF-κB, CDX-2, and BMP-4 signaling pathways by COX-2 may be part of this mechanism. In the process of BE occurrence and heteromorphism transformation, acid, bile salt, and their mixture play a specific role, and the mixture of these two displayed the strongest effect. In the clinical treatment of BE and GERD, we should not only pay attention to the role of acid, but also to the presence of bile reflux.

## Supplementary Information

**Additional file 1.** 

## Data Availability

All data generated or analyzed during this study are included in this published article. The datasets used and/or analyzed during the current study are available from the corresponding author on reasonable request.

## Abbreviations

BE: Barrett’s esophagus
SQ: Squamous epithelium
GERD: Gastroesophageal reflux disease
EAC: Esophageal adenocarcinoma
COX-2: Cyclooxygenase-2
HET-1A: Esophageal squamous cell line
BAR-T: Barrett esophageal cell line
NF-Κb: Nuclear factor kappa B
BMP-4: Bone morphogenetic protein-4
CDX-2: Caudal-related homeobox transcription factor-2
TEM: Transmission electron microscopy
