# Supplementary material for: COX-2 strengthens the effects of acid and bile salts on human esophageal cells and Barrett esophageal cells
Source: BMC Mol Cell Biol. 2022 Apr 12;23:19. doi: 10.1186/s12860-022-00418-5 (PMC9004192; doi:10.1186/s12860-022-00418-5)

**FIG.1**

| **HET-1A** |  |  |  | **BAR-T** |  |  |  |
| --- | --- | --- | --- | --- | --- | --- | --- |
| **DAY1** |  |  |  | **DAY1** |  |  |  |
| **NC** | 0.360 | 0.363 | 0.343 | **NC** | 0.300 | 0.322 | 0.343 |
| **COX-2** | 0.321 | 0.322 | 0.326 | **COX-2** | 0.333 | 0.330 | 0.329 |
| **si NC** | 0.363 | 0.379 | 0.367 | **si NC** | 0.316 | 0.309 | 0.306 |
| **si COX-2** | 0.373 | 0.367 | 0.349 | **si COX-2** | 0.322 | 0.327 | 0.310 |
|  |  |  |  |  |  |  |  |
| **DAY2** |  |  |  | **DAY2** |  |  |  |
| **NC** | 0.559 | 0.545 | 0.560 | **NC** | 0.533 | 0.541 | 0.550 |
| **COX-2** | 0.612 | 0.623 | 0.615 | **COX-2** | 0.619 | 0.618 | 0.592 |
| **si NC** | 0.769 | 0.750 | 0.761 | **si NC** | 0.564 | 0.561 | 0.586 |
| **si COX-2** | 0.679 | 0.667 | 0.664 | **si COX-2** | 0.512 | 0.488 | 0.494 |
|  |  |  |  |  |  |  |  |
| **DAY3** |  |  |  | **DAY3** |  |  |  |
| **NC** | 0.751 | 0.740 | 0.750 | **NC** | 0.621 | 0.613 | 0.623 |
| **COX-2** | 0.811 | 0.817 | 0.804 | **COX-2** | 0.692 | 0.678 | 0.691 |
| **si NC** | 0.823 | 0.815 | 0.822 | **si NC** | 0.678 | 0.666 | 0.675 |
| **si COX-2** | 0.774 | 0.763 | 0.762 | **si COX-2** | 0.628 | 0.613 | 0.626 |

**FIG.2**


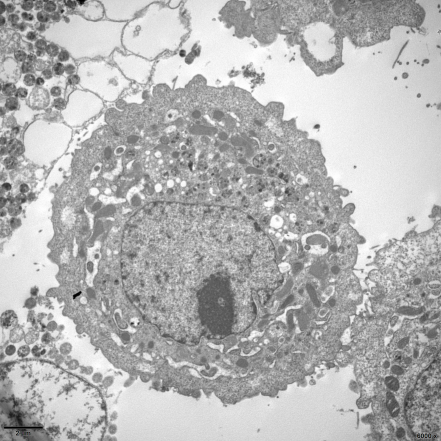

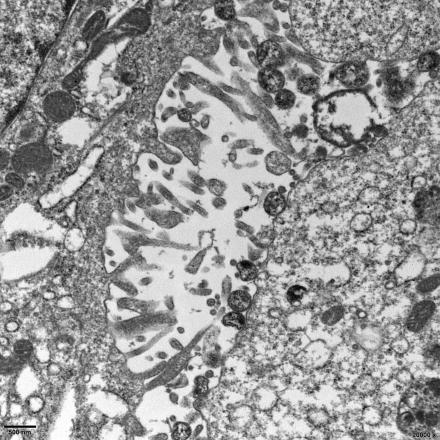

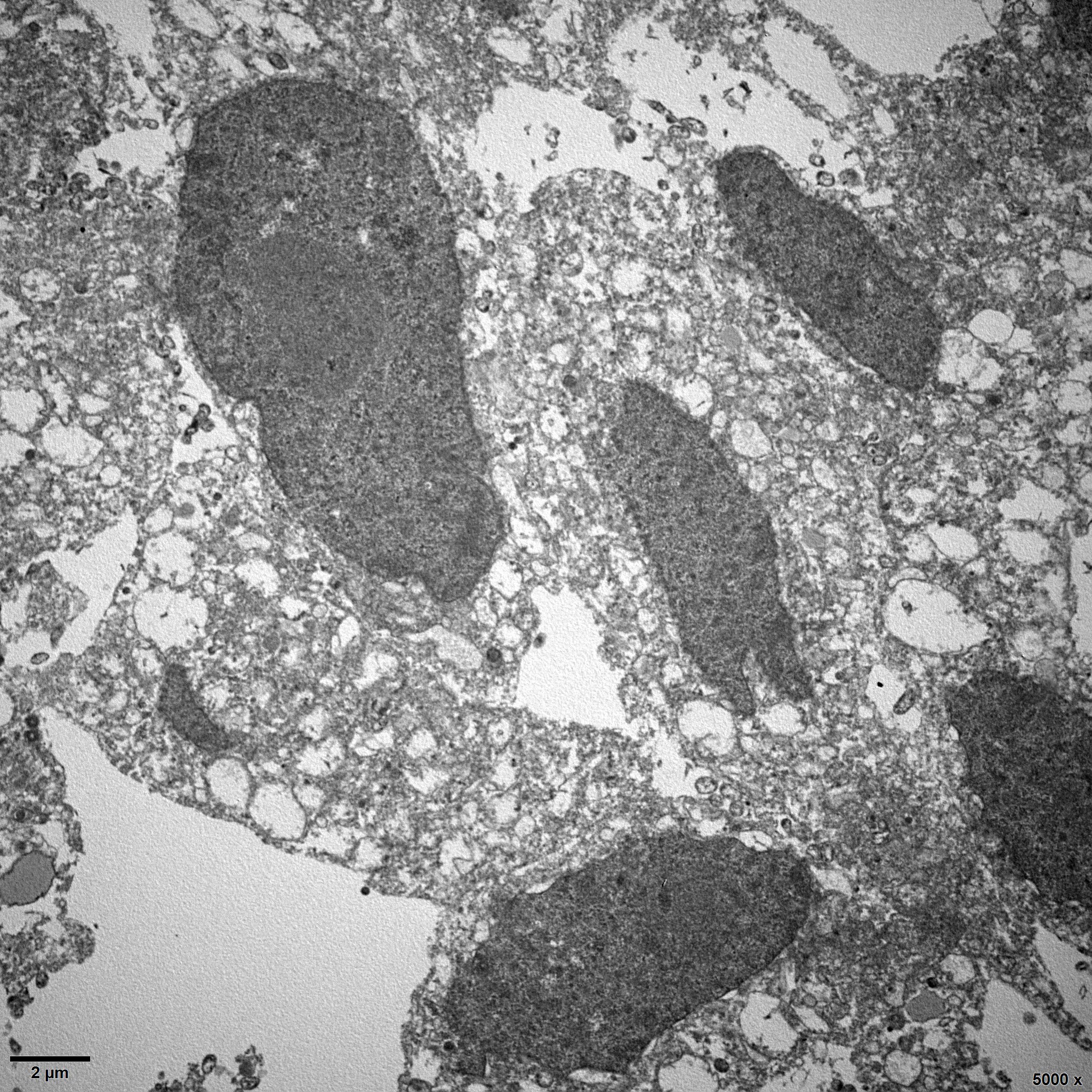

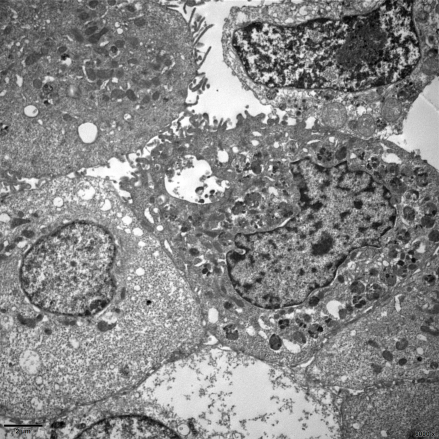

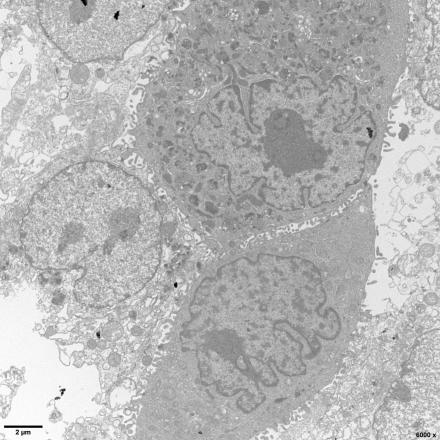

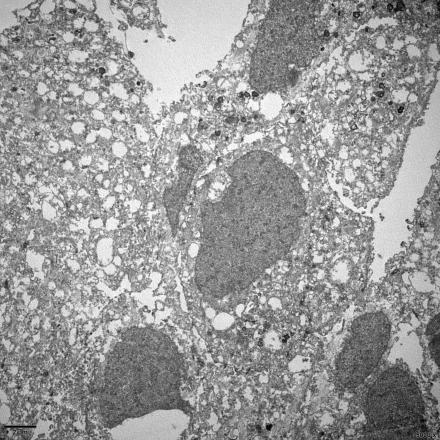


**FIG.3**

| HET-1A | NC | | | COX-2 | | | SiNC | | | SiCOX-2 | | |
| --- | --- | --- | --- | --- | --- | --- | --- | --- | --- | --- | --- | --- |
| COX2 | 0.823 | 0.891 | 0.801 | 0.956 | 0.982 | 0.973 | 0.335 | 0.317 | 0.378 | 0.053 | 0.039 | 0.026 |
| MUC2 | 0.722 | 0.739 | 0.75 | 0.707 | 0.712 | 0.723 | 0.562 | 0.435 | 0.495 | 0.723 | 0.716 | 0.731 |
| c-myb | 0.286 | 0.307 | 0.282 | 0.259 | 0.313 | 0.287 | 0.335 | 0.301 | 0.291 | 0.223 | 0.309 | 0.273 |
| CDX2 | 0.223 | 0.216 | 0.202 | 0.335 | 0.36 | 0.359 | 0.412 | 0.493 | 0.487 | 0.197 | 0.219 | 0.183 |
| BMP4 | 0.425 | 0.473 | 0.456 | 0.633 | 0.657 | 0.662 | 0.456 | 0.526 | 0.503 | 0.172 | 0.238 | 0.239 |
| p-P65 | 0.533 | 0.562 | 0.517 | 0.783 | 0.732 | 0.75 | 0.176 | 0.209 | 0.211 | 0.005 | 0.08 | 0.1 |
| P65 | 0.787 | 0.793 | 0.782 | 0.753 | 0.739 | 0.746 | 0.722 | 0.761 | 0.775 | 0.735 | 0.801 | 0.79 |
|  |  |  |  |  |  |  |  |  |  |  |  |  |
|  |  |  |  |  |  |  |  |  |  |  |  |  |
| BAR-T | NC |  |  | COX-2 |  |  | SiNC |  |  | SiCOX-2 |  |  |
| COX2 | 0.425 | 0.496 | 0.433 | 0.653 | 0.68 | 0.673 | 0.367 | 0.355 | 0.398 | 0.213 | 0.237 | 0.231 |
| MUC2 | 0.321 | 0.337 | 0.356 | 0.337 | 0.312 | 0.353 | 0.262 | 0.234 | 0.295 | 0.32 | 0.316 | 0.333 |
| c-myb | 0.086 | 0.017 | 0.082 | 0.059 | 0.063 | 0.088 | 0.035 | 0.061 | 0.091 | 0.123 | 0.079 | 0.073 |
| CDX2 | 0.123 | 0.116 | 0.107 | 0.135 | 0.137 | 0.159 | 0.112 | 0.196 | 0.189 | 0.043 | 0.019 | 0.035 |
| BMP4 | 0.125 | 0.175 | 0.158 | 0.483 | 0.456 | 0.562 | 0.456 | 0.526 | 0.503 | 0.171 | 0.223 | 0.231 |
| p-P65 | 0.333 | 0.302 | 0.319 | 0.782 | 0.739 | 0.752 | 0.776 | 0.703 | 0.722 | 0.107 | 0.138 | 0.177 |
| P65 | 0.732 | 0.715 | 0.725 | 0.757 | 0.730 | 0.796 | 0.782 | 0.766 | 0.795 | 0.775 | 0.803 | 0.759 |

**FIG.4A**

| **HET-1A** | **4000个/孔** |  |  |  |  |
| --- | --- | --- | --- | --- | --- |
| 0uM | 0.619 | 0.589 | 0.646 | 0.644 | 0.633 |
| 400uM | 0.621 | 0.657 | 0.659 | 0.664 | 0.656 |
| 800uM | 0.556 | 0.517 | 0.530 | 0.562 | 0.536 |
| 1200uM | 0.246 | 0.241 | 0.244 | 0.245 | 0.243 |
|  |  |  |  |  |  |
| **BAR-T** | **4000个/孔** |  |  |  |  |
| 0uM | 0.470 | 0.460 | 0.476 | 0.480 | 0.460 |
| 800uM | 0.444 | 0.444 | 0.452 | 0.445 | 0.445 |
| 1200uM | 0.301 | 0.303 | 0.308 | 0.304 | 0.301 |
| 1600uM | 0.224 | 0.210 | 0.214 | 0.215 | 0.216 |

**FIG.4B**

| 4000个/孔 | 10%MTS孵育2h |  |  |  |  |
| --- | --- | --- | --- | --- | --- |
| HET-1A |  |  |  |  |  |
| 1200uM 0min | 1.225 | 1.267 | 1.226 | 1.226 | 1.216 |
| 1200uM 30min | 0.834 | 0.860 | 0.837 | 0.864 | 0.856 |
| 1200uM 60min | 0.557 | 0.565 | 0.590 | 0.559 | 0.553 |
| 1200uM 90min | 0.433 | 0.450 | 0.431 | 0.458 | 0.443 |
|  |  |  |  |  |  |
| BAR-T |  |  |  |  |  |
| 1200uM 0min | 0.563 | 0.560 | 0.556 | 0.557 | 0.565 |
| 1200uM 30min | 0.497 | 0.492 | 0.494 | 0.488 | 0.510 |
| 1200uM 60min | 0.438 | 0.421 | 0.403 | 0.425 | 0.441 |
| 1200uM 90min | 0.371 | 0.368 | 0.357 | 0.332 | 0.369 |

**FIG.4C**

**



**

| HET-1A |  |  | BAR-T |  |
| --- | --- | --- | --- | --- |
| 0 | 0.621 |  | 0 | 0.103 |
|  | 0.635 |  |  | 0.117 |
|  | 0.618 |  |  | 0.091 |
| 30 | 0.715 |  | 30 | 0.125 |
|  | 0.723 |  |  | 0.176 |
|  | 0.708 |  |  | 0.122 |
| 60 | 0.789 |  | 60 | 0.281 |
|  | 0.802 |  |  | 0.237 |
|  | 0.792 |  |  | 0.201 |
| 90 | 0.805 |  | 90 | 0.334 |
|  | 0.817 |  |  | 0.305 |
|  | 0.813 |  |  | 0.316 |

**FIG.5A**

| HET-1A | 4000个/孔 | 10%MTS孵育1h |  |  |  |
| --- | --- | --- | --- | --- | --- |
| NC | 1.010 | 0.963 | 0.958 | 0.968 | 0.963 |
| PH 4.0 | 0.433 | 0.432 | 0.439 | 0.400 | 0.393 |
| PH 5.0 | 0.787 | 0.788 | 0.803 | 0.806 | 0.797 |
| PH 6.0 | 0.764 | 0.773 | 0.746 | 0.763 | 0.743 |
|  |  |  |  |  |  |
| BAR-T |  |  |  |  |  |
| NC | 0.665 | 0.624 | 0.620 | 0.668 | 0.644 |
| PH 4.0 | 0.321 | 0.294 | 0.298 | 0.296 | 0.300 |
| PH 5.0 | 0.600 | 0.595 | 0.646 | 0.589 | 0.624 |
| PH 6.0 | 0.621 | 0.641 | 0.638 | 0.634 | 0.631 |

**

FIG.5B**

**

**

| HET-1A |  |  | BAR-T |  |
| --- | --- | --- | --- | --- |
| 7 | 0.322 |  | 7 | 0.403 |
|  | 0.305 |  |  | 0.416 |
|  | 0.318 |  |  | 0.431 |
| 6 | 0.252 |  | 6 | 0.325 |
|  | 0.273 |  |  | 0.306 |
|  | 0.208 |  |  | 0.318 |
| 5 | 0.181 |  | 5 | 0.181 |
|  | 0.206 |  |  | 0.151 |
|  | 0.195 |  |  | 0.13 |
| 4 | 0.135 |  | 4 | 0.125 |
|  | 0.117 |  |  | 0.097 |
|  | 0.167 |  |  | 0.106 |

**

**

**FIG.6**

| 4000个/孔 | 10%MTS孵育2h |  |  |  |
| --- | --- | --- | --- | --- |
| 0.594 | 0.589 | 0.580 | 0.593 | 0.559 |
| 0.409 | 0.417 | 0.412 | 0.421 | 0.414 |
| 0.434 | 0.423 | 0.438 | 0.424 | 0.427 |
| 0.389 | 0.397 | 0.398 | 0.382 | 0.384 |
|  |  |  |  |  |
|  |  |  |  |  |
| 0.493 | 0.483 | 0.493 | 0.508 | 0.507 |
| 0.357 | 0.391 | 0.417 | 0.387 | 0.389 |
| 0.464 | 0.458 | 0.453 | 0.452 | 0.462 |
| 0.197 | 0.196 | 0.201 | 0.198 | 0.197 |

**
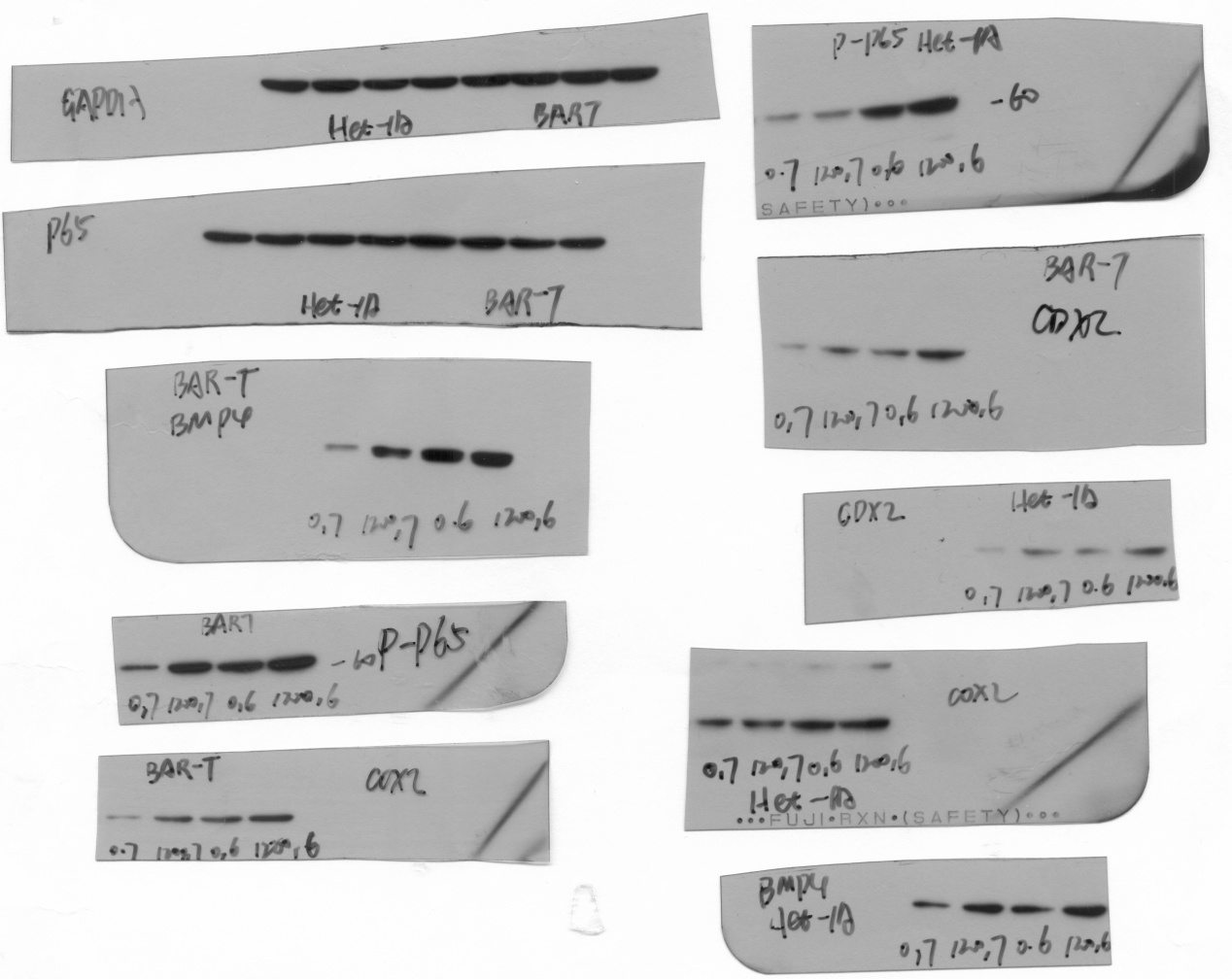
FIG.7**

| HET-1A | COX2 | CDX2 | BMP4 | p-P65 | P65 |
| --- | --- | --- | --- | --- | --- |
| 0μM PH7.0 | 0.527 | 0.037 | 0.423 | 0.135 | 0.983 |
|  | 0.592 | 0.055 | 0.477 | 0.164 | 0.95 |
|  | 0.521 | 0.058 | 0.461 | 0.119 | 0.985 |
| 1200μM PH7.0 | 0.652 | 0.139 | 0.723 | 0.183 | 0.953 |
|  | 0.684 | 0.126 | 0.688 | 0.192 | 0.939 |
|  | 0.677 | 0.117 | 0.762 | 0.209 | 0.996 |
| 0μM PH6.0 | 0.835 | 0.121 | 0.653 | 0.876 | 0.957 |
|  | 0.839 | 0.138 | 0.628 | 0.853 | 0.964 |
|  | 0.876 | 0.117 | 0.702 | 0.882 | 0.975 |
| 1200μM PH6.0 | 0.952 | 0.293 | 0.932 | 0.905 | 0.955 |
|  | 0.932 | 0.263 | 0.938 | 0.978 | 0.981 |
|  | 0.923 | 0.298 | 0.934 | 0.967 | 0.979 |

| BAR-T | COX2 | CDX2 | BMP4 | p-P65 | P65 |
| --- | --- | --- | --- | --- | --- |
| 0μM PH7.0 | 0.122 | 0.063 | 0.153 | 0.193 | 0.98 |
|  | 0.116 | 0.079 | 0.167 | 0.196 | 0.965 |
|  | 0.13 | 0.053 | 0.177 | 0.187 | 0.966 |
| 1200μM PH7.0 | 0.253 | 0.175 | 0.585 | 0.885 | 0.967 |
|  | 0.218 | 0.153 | 0.536 | 0.832 | 0.948 |
|  | 0.275 | 0.157 | 0.551 | 0.853 | 0.996 |
| 0μM PH6.0 | 0.364 | 0.162 | 0.872 | 0.771 | 0.982 |
|  | 0.359 | 0.181 | 0.866 | 0.706 | 0.973 |
|  | 0.338 | 0.182 | 0.823 | 0.728 | 0.995 |
| 1200μM PH6.0 | 0.516 | 0.746 | 0.959 | 0.935 | 0.972 |
|  | 0.532 | 0.717 | 0.983 | 0.943 | 0.983 |
|  | 0.558 | 0.733 | 0.972 | 0.956 | 0.959 |

**FIG.8**

| HET-1A | 4000个/孔 | 10%MTS孵育1h |  |  |
| --- | --- | --- | --- | --- |
| HET-1A | 0.387 | 0.394 | 0.4 | 0.396 |
| siNC+1200uM+PH6.0 | 0.235 | 0.236 | 0.244 | 0.236 |
| siCOX2+1200uM+PH6.0 | 0.239 | 0.2 | 0.215 | 0.21 |
|  |  |  |  |  |
|  |  |  |  |  |
| BAR-T |  |  |  |  |
| BAR-T | 0.341 | 0.341 | 0.328 | 0.357 |
| siNC+1200uM+PH6.0 | 0.227 | 0.235 | 0.224 | 0.238 |
| siCOX2+1200uM+PH6.0 | 0.199 | 0.197 | 0.195 | 0.197 |

**FIG.9**

**
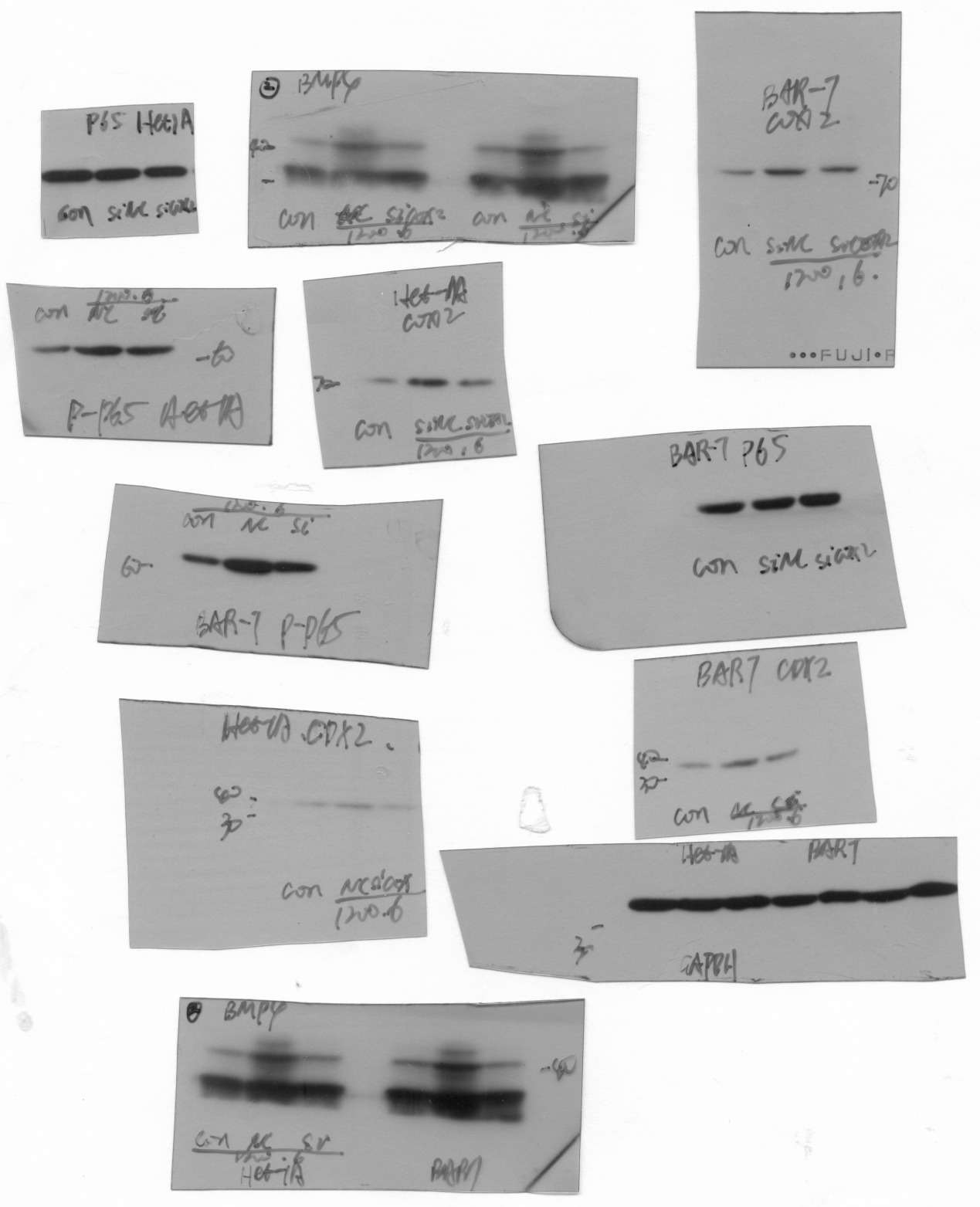
**

| HET-1A | | COX2 | | CDX2 | | BMP4 | | p-P65 | | P65 | |
| --- | --- | --- | --- | --- | --- | --- | --- | --- | --- | --- | --- |
| CON | | 0.087 | | 0.056 | | 0.222 | | 0.297 | | 0.982 | |
|  |  | 0.102 | | 0.063 | | 0.243 | | 0.316 | | 0.973 | |
|  |  | 0.922 | | 0.05 | | 0.219 | | 0.315 | | 0.975 | |
| SiNC | | 0.725 | | 0.131 | | 0.523 | | 0.585 | | 0.957 | |
|  |  | 0.738 | | 0.128 | | 0.627 | | 0.531 | | 0.968 | |
|  |  | 0.709 | | 0.113 | | 0.566 | | 0.557 | | 0.972 | |
| SiCOX-2 | | 0.332 | | 0.026 | | 0.193 | | 0.471 | | 0.963 | |
|  |  | 0.313 | | 0.039 | | 0.202 | | 0.408 | | 0.975 | |
|  |  | 0.296 | | 0.038 | | 0.197 | | 0.436 | | 0.986 | |
| BAR-T | COX2 | | CDX2 | | BMP4 | | p-P65 | | P65 | |  |
| CON | 0.152 | | 0.062 | | 0.073 | | 0.497 | | 0.971 | |  |
|  | 0.162 | | 0.079 | | 0.085 | | 0.473 | | 0.963 | |  |
|  | 0.536 | | 0.058 | | 0.076 | | 0.458 | | 0.955 | |  |
| SiNC | 0.456 | | 0.195 | | 0.481 | | 0.985 | | 0.979 | |  |
|  | 0.413 | | 0.183 | | 0.439 | | 0.976 | | 0.965 | |  |
|  | 0.478 | | 0.207 | | 0.453 | | 0.955 | | 0.967 | |  |
| SiCOX-2 | 0.304 | | 0.159 | | 0.17 | | 0.67 | | 0.973 | |  |
|  | 0.315 | | 0.161 | | 0.165 | | 0.654 | | 0.968 | |  |
|  | 0.327 | | 0.173 | | 0.172 | | 0.639 | | 0.983 | |  |

**FIG.10**


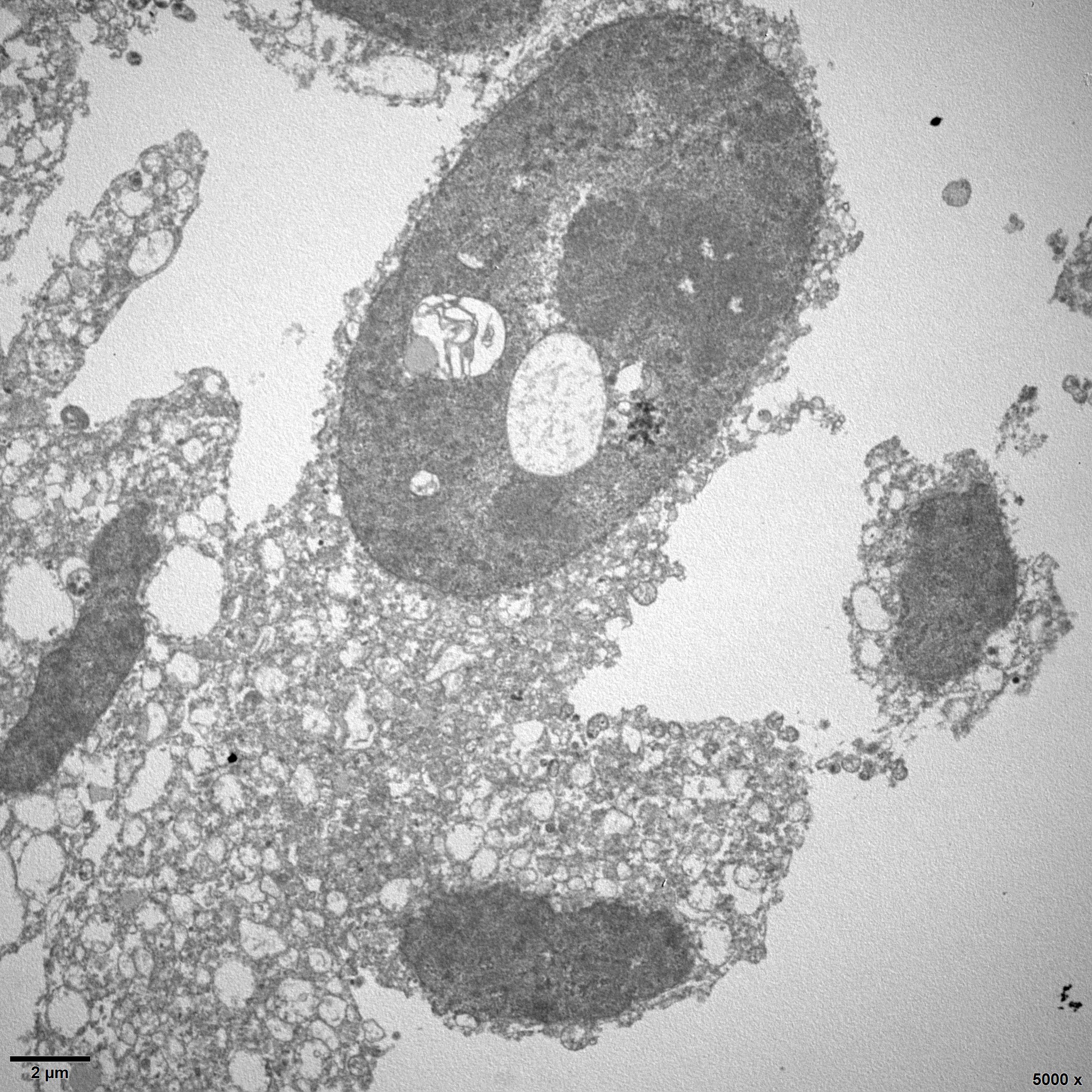

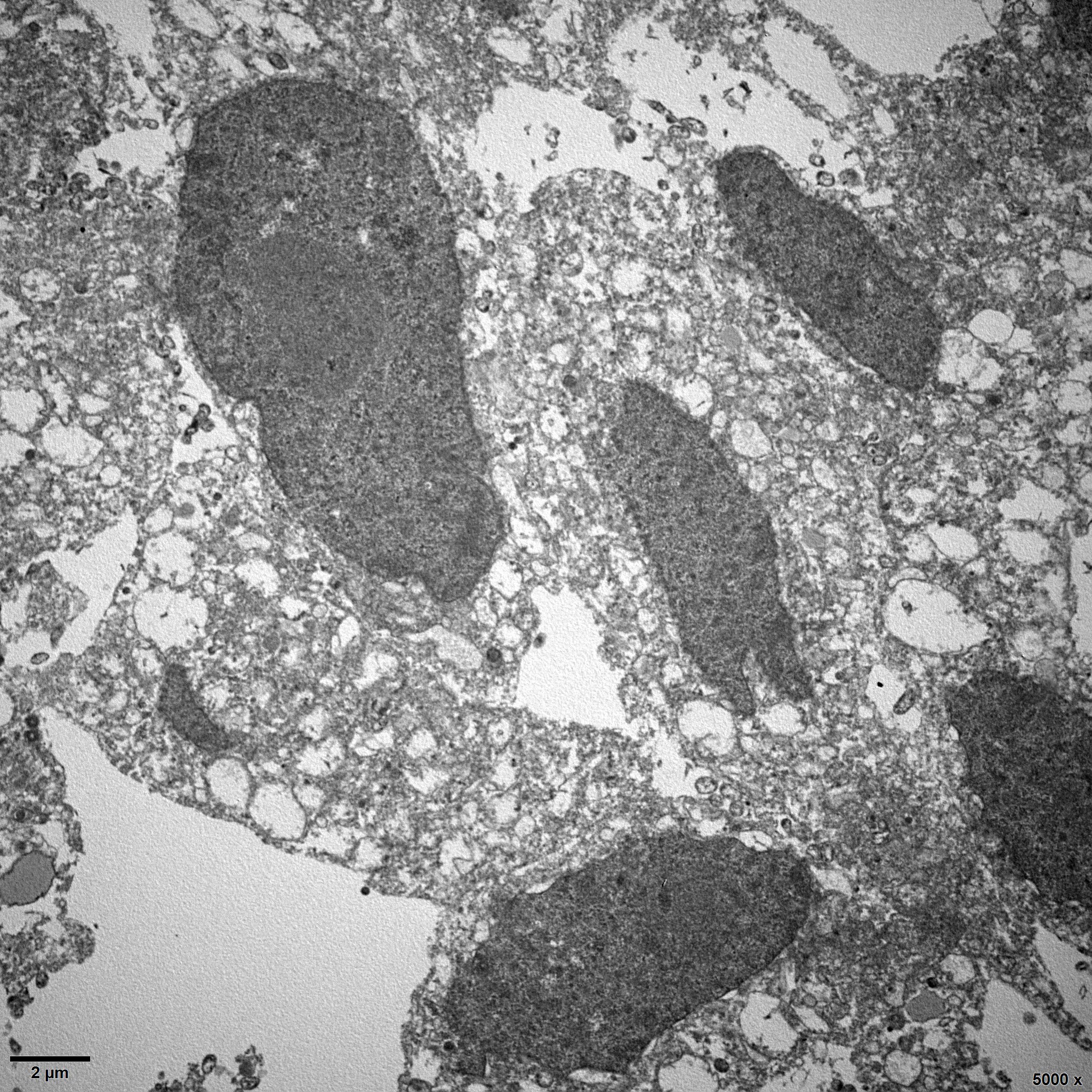

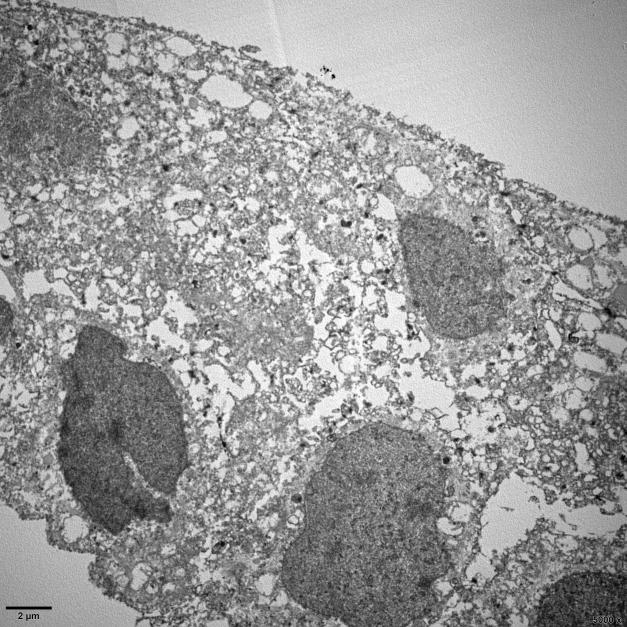

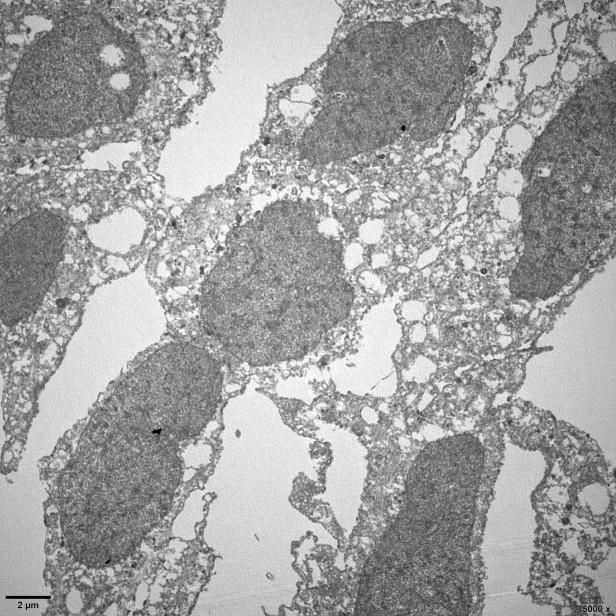

Supplement: Supplementary file 1 — Additional file 1. [file 12860_2022_418_MOESM1_ESM.docx]
